# Dietary berberine supplementation improves select physiological and psychological responses during exertional heat stress: A pilot study in young adults

**DOI:** 10.14814/phy2.70937

**Published:** 2026-05-27

**Authors:** Dante A. Van Arman, Jacob C. Saunders, Yaw O. Korankyi, Emerson P. Heckler, Ben J. Lee, Trevor L. Gillum, Matthew R. Kuennen

**Affiliations:** ^1^ Department of Exercise Science High Point University High Point North Carolina USA; ^2^ Occupational and Thermal Physiology, Centre for Physical Activity, Sport and Exercise Sciences Coventry University Coventry UK; ^3^ Department of Kinesiology California Baptist University Riverside California USA

**Keywords:** berberine, cardiovascular, dietary supplement, exercise, heat stress, pulmonary, thermoregulation

## Abstract

Berberine has been shown to lower core temperature and heart rate during passive heat stress in animal models. This study evaluated the effect of dietary berberine supplementation on physiological and perceptual responses to exercise‐heat stress in humans. Eight participants (6 men/2 women; Age: 23 ± 3 years, Stature: 1.75 ± 0.03 m, Mass: 71.8 ± 2.7 kg, VO_2max_: 57.5 ± 2.1 mL/kg lbm/min^−1^) ingested 1.5 g of Berberine or Placebo for 7d prior to a 1 h treadmill run (60% VO_2max_) in hot (35°C), moderately humid (39% RH) conditions. Mean body temperature (T_b_), heart rate (HR), expired gasses (VO_2_, VCO_2_, RER), minute ventilation, respiratory rate (R_R_), tidal volume, and perceptual responses (thermal sensation, perceived discomfort, and perceived exertion) were measured throughout exercise. Data are reported as average ± standard deviation over the 60 min exercise trial. T_b_ was lower (p = 0.030) in Berberine (37.69 ± 0.53°C) than Placebo (37.84 ± 0.38°C). Heart rate was lower (p = 0.025) in Berberine (163 ± 28 bpm) than Placebo (166 ± 30 bpm). R_R_ was lower (p = 0.045) in Berberine (38.8 ± 11.2 bpm) than Placebo (41.4 ± 10.6 bpm). Thermal sensation was lower (*p* = 0.002) in Berberine (9.5 ± 4.2) than Placebo (12.3 ± 5.0). Generalized discomfort was lower (*p* = 0.044) in Berberine (9.3 ± 5.1) than Placebo (12.1 ± 5.6). Perceived exertion was lower (*p* < 0.001) in Berberine (11.4 ± 3.4) than Placebo (13.1 ± 4.0). Dietary berberine supplementation improved mean body temperature, heart rate, respiratory rate, and perceptual responses during exertional heat stress. Many of these changes were of small magnitude, calling into question the potential utility of dietary berberine supplementation for work or exercise in hot ambient conditions.

## 1 INTRODUCTION

Prolonged work or exercise in hot, humid conditions increases core temperature and reduces total body water (Rowell, 1974), causing cardiovascular strain (Kuennen et al., 2011) that promotes hepatosplanchinc shunting (Rowell et al., 1964) and gastrointestinal barrier damage (Bouchama et al., 2022). This allows gram‐negative bacteria to translocate from the gut into circulation, increasing circulating concentrations of lipopolysaccharide (LPS) and activating NF‐κB mediated pro‐inflammatory cascades (Kuennen et al., 2011). Regular work or exercise does not necessarily induce such catastrophic responses. However, in the worst‐case scenario where core temperature exceeds 41°C, these changes can trigger disseminated intravascular coagulation (DIC), necrosis of organ tissues, multi‐organ failure, and ultimately death (Bouchama et al., 2022; Lim & Mackinnon, 2006).

Over the past decade our group has examined multiple dietary supplements that may help improve outcomes during exertional heat stress (Conrad et al., 2024; Falgiano et al., 2018; Flood et al., 2020; Hiles et al., 2020; Kuennen et al., 2015; Lee et al., 2022; McKenna et al., 2020; Szymanski et al., 2018). Recently we became interested in berberine, a plant‐derived isoquinoline quaternary alkaloid that has been a core component of traditional Chinese and Indian herbal medicine for centuries (Zou et al., 2017). Berberine is commonly used to treat gastrointestinal infections and has been shown to reduce intravascular coagulation in bacterial sepsis (Yuan et al., 2021), affording beneficial effects in the lungs (Izadparast et al., 2022; Tew et al., 2020) and livers (Sahin et al., 2013) of animals that were challenged with LPS or passive heat stress. Pretreatment with intragastric or intraperitoneal berberine reduced febrile response in rabbits that were challenged with LPS (Chu et al., 2014). It also reduced heart rate and core temperature in mice that were challenged with passive heat stress (40°C) (Jiang et al., 2013), which the authors mechanistically attributed to a suppression of feed‐forward thermal afferent signaling via hypothalamic thermosensitive neurons. Berberine has also been shown to increase calcium levels in isolated myocardial cells (Shaffer, 1985), producing an inotropic effect that improves hemodynamic responses (cardiac index and left ventricular ejection fraction) in congestive heart failure patients (Marin‐Neto et al., 1988). In the context of exertional heat stress, improved inotropic function might help to better maintain cardiac output and skin blood flow, improving heat dissipation. Berberine also directly activates AMP‐activated protein kinase (AMPK), where phosphorylation of AMPK has been shown to increase fatty acid uptake and oxidation as well as improve mitochondrial function and ATP production (Habiba et al., 2025). Greater reliance on lipid metabolism could contribute to lower heat production during exertional heat stress. Interestingly, berberine‐mediated activation of AMPK has also been tied to reduced anxiety in a rat model of colitis (Habiba et al., 2025) and resolution of depression‐like behavior in mice that were challenged with chronic restraint stress (Cheng et al., 2025). Further, low dose administration of berberine (5 mg/kg i.p.) in mice was shown to increase circulating concentrations of norepinephrine (NE) and serotonin (5‐HT), leading to improvements in motivation and mood status (Fan et al., 2019). If translated to a human exercise model, these benefits could improve participants' perceptions of exertional heat stress.

Despite these known benefits on thermoregulatory and cardiovascular function as well as potential benefits on mood status, the impact of dietary berberine supplementation on exertional heat stress responses in humans has not been previously examined. Therefore, the purpose of this study was to examine the effects of short term (1 week) dietary berberine supplementation (1.5 g/d) on systems‐level physiology parameters (V_E_, VO_2_, VCO_2_, RER, T_c_, T_sk_, T_b_, HR, PSI, hydration), perceptual responses (ratings of thermal sensation, generalized discomfort, and perceived exertion), and select blood markers of motivation (NE, 5‐HT) in participants that were challenged with 60 min of submaximal treadmill exercise (60% VO_2max_) under hot (35°C) and moderately humid (39% RH) ambient conditions. Based on prior outcomes in animal models, it was hypothesized that berberine would reduce heart rate and core temperature responses, resulting in improved perceptual responses during exercise‐heat stress.

## 2 MATERIALS AND METHODS

### 2.1 Participants

Eight recreationally active participants (6 men and 2 women; Age: 23 ± 3 years, Stature: 1.75 ± 0.03 m, Mass: 71.8 ± 2.7 kg, VO_2max_: 57.5 ± 2.1 mL·kg·lbm·min^−1^) provided written informed consent prior to completing a double‐blind placebo‐controlled study with randomized crossover design. All data were collected at High Point University (High Point, NC, USA) where participants were recruited by fliers posted around the campus and by word of mouth. All participants were nonsmokers, normotensive, and did not have cardiovascular, pulmonary, or metabolic disease as defined by the American College of Sports Medicine (Riebe et al., 2015). They were also recreationally active and did not disclose any history of heat illness, GI barrier dysfunction, or current medication/nutritional supplement utilization that could influence study outcomes. Data were collected in summer months and participants were asked to maintain their current exercise regimen for study duration. Each participant in this study completed two identical bouts of treadmill exercise. One bout was performed following a 7d regimen of dietary berberine supplementation (1.5 g/d) and the other following a 7d regimen of placebo supplementation. Condition order was counterbalanced, supplementation was double‐blind, and a minimum of a 2‐week washout period was provided between study conditions. One female participant was taking a monophasic oral contraceptive. Both female participants were eumenorrheic and primary study data were collected in the follicular phase following menstruation. Study procedures were approved by the ethics committee of High Point University (High Point, NC, USA) and were in accord with the Declaration of Helsinki.

### 2.2 Preliminary assessment

Prior to data collection, all participants underwent body composition analysis via three site skinfolds (Male: chest, abdomen, and thigh; Female: tricep, suprailiac, and thigh). Body density was attained through duplicate measures at each site with the values summed and incorporated into a standardized regression equation. Body density was then used to estimate body composition (Brozek et al., 1963). A graded treadmill test was used to assess maximal aerobic capacity (VO_2max_). VO_2max_ was determined using open‐circuit calorimetry (TrueOne 2400, ParvoMedics, Salt Lake City, UT) and was defined as the highest 10s value when two of the following conditions were met: (1) a plateau in VO_2_ (change in VO_2_ < 150 mL/min) with increased workload, (2) a maximal respiratory exchange ratio greater than 1.1, and (3) heart rate greater than 90% of the age predicted maximum (Gaskill et al., 2023). After the VO_2max_ test, participants completed three 5‐min bouts of submaximal exercise to identify the treadmill speed that would elicit 60% of their individual VO_2max_. They used this same workload in both of their experimental trials.

### 2.3 Study diet and dietary supplement

Participants used standard food diaries to record their dietary intake for 48 h prior to their first experimental visit. Upon completion of their first experimental visit, participants were given a copy of their food log and asked to replicate this dietary intake prior to their second visit. Dietary intake was quantified using publicly available software (USDA National Nutrient Database for Standard Reference). Berberine is not found in any common food sources and is not a normal component of the western diet. However, dietary intake was examined to ensure natural sources of berberine (barberry, tree turmeric, Oregon grape, and goldenseal) were not present in the diet. For 1 week prior to each of the two experimental visits, participants consumed 1.5 g of berberine (>99% purity; Source: PureBulk, Roseburg, OR, USA) or 1.5 g of maltodextrin (Source: Nutricost, Vineyard, UT, USA). The dietary supplement and placebo were provided in 2 opaque capsules and participants were instructed to ingest 1 capsule with their morning and evening meals. Participants ingested their last dose of berberine or placebo approximately 1 h prior to their exercise testing session.

This dose and twice daily dosing strategy were adopted based on recommendations from a meta‐analysis of prior human intervention studies (Dong et al., 2012). That report outlines that unlike prior work in animal models, in human subjects research berberine intake is normally prescribed as an absolute value (ranging between 0.5 g and 1.5 g per day) and provided in multiple doses throughout the day (Dong et al., 2012). Plasma berberine concentrations were not measured in the present study. However, we can make inferences based on prior research. For example, a pharmacokinetic study that examined 20 human participants following a single 400 mg oral dose of berberine identified a maximal plasma concentration of 0.4 ng/mL (Hua et al., 2007). Another study reported that plasma berberine concentrations were increased to 1.2 ± 0.4 ng/mL following 3 months of 900 mg/d oral berberine supplementation, which represented a 5.8 fold increase compared with berberine pretreatment (Xu et al., 2020). Based on the results of these studies, we estimate that our dosing strategy (1.5 g dietary berberine supplementation ingested over 2 daily doses for 1 week) would result in plasma concentrations of 1.5 ng/mL. Since oral berberine supplementation is commonly used to improve blood glucose regulation, promote weight loss, and reduce other cardiovascular disease risk factors, prior human subjects research has commonly utilized extended supplementation periods (≥8 weeks) to allow adequate time to detect changes in those primary outcome measures (Zamani et al., 2022). In contrast, prior work in animal models that examined berberine's impact on core temperature regulation utilized a single berberine dose prior to stress exposure (Chu et al., 2014; Jiang et al., 2013; Yuan et al., 2021). Since the impact of oral berberine supplementation on core temperature regulation had not been examined during exercise or in humans, we were concerned that one single dose may not impart adequate effects. For that reason, we utilized a 1 week supplementation period, which has been successfully employed by our group and others in prior dietary supplement research (Conrad et al., 2024; Zuhl et al., 2014).

Berberine has a distinctive yellow color and it was possible that participants could have attempted to open their opaque capsules to determine which supplement they were receiving. For that reason powdered food coloring (Iberia Flavors Yellow Coloring, Miami, FL, USA) was used to ensure the berberine and maltodextrin powders were visually identical. Blinding was successful, as indicated by five of eight participants incorrectly guessing the order of supplements they received. Blinding was not broken until after all study data were collected. Experimental conditions were separated by a 14‐day washout period, which is appropriate because the expected half‐life of berberine in circulation is ≤12 h. Trial order was determined using a free online tool (https://www.randomizer.org) and four participants received berberine as the first condition. All experimental trials were conducted in an environmental chamber controlled at 35°C and 39.3% RH.

### 2.4 Experimental protocol

Prior to testing, participants were asked to refrain from the following: (1) exercise 48 h prior to the exercise trial; (2) alcohol and caffeine 24 h prior to testing; and (3) food consumption 8 h prior to testing (overnight fast). On the morning of each experimental trial, participants were instructed to drink ~400 mL of water and arrive at the laboratory between 08:00 and 10:00. At that time a blood sample was taken, the procedure for which is described below. A urine sample was also taken for assessment of urine specific gravity (REF312ATC; General Tools & Instruments, New York, NY, USA), to ensure participants were euhydrated (USG ≤ 1.025) (Sawka et al., 2007). Body height and mass were quantified without shoes and after participants had voided their bladder. Dressed in standard athletic clothing (shorts, sports bra or t‐shirt, athletic socks, and running shoes), participants next inserted a calibrated thermistor (YSI Precision 440 Series, Yellow Springs Inc., Yellow Springs, OH, USA) into the esophagus to a depth of one quarter of body height. Uncovered skin thermistors (Grant Instruments Ltd., Cambridge, UK) were attached to the mid‐belly of the pectoralis major, triceps brachii, rectus femoris, and gastrocnemius for calculation of mean skin temperature (Ramanathan, 1964). Esophageal and skin thermistors integrated with a data logger (SQ 2040; Grant Instruments Ltd., Cambridge, UK) that recorded core (T_c_) and skin (T_sk_) temperatures at 15 s intervals. From these, mean body temperature (T_b_) was calculated using a standard equation (Kenney, 1998), and all temperature data are reported at 5 min intervals.

Participants were also fitted with a standard radio telemetry strap (RS400sd; Polar Instruments, Kempele, Finland) for heart rate (HR) assessment and a noseclip and mouthpiece for expired gas analysis (TrueOne 2400, ParvoMedics, Salt Lake City, UT). They remained stationary on the treadmill for 5 min, during which time baseline readings of oxygen consumption (VO_2_), carbon dioxide production (VCO_2_), respiratory exchange (RER), respiratory rate (R_R_), tidal volume (V_T_), and minute ventilation (V_E_), HR, T_c_, and T_sk_ were taken. Participants were also asked to provide ratings of perceived exertion (Borg, 1970), thermal sensation and generalized discomfort (Gagge et al., 1969) during this period, and at 5 min intervals throughout exercise. The thermal sensation and generalized discomfort scales were modified from their original format for use in hot ambient conditions as described previously (Kuennen et al., 2015), according to the procedures outlined by Zhang (2003). For thermal sensation, 0 = slightly cool, 4 = neutral, 8 = slightly warm, 12 = warm, 16 = hot, and 20 = very hot. For generalized discomfort, 0 = very comfortable, 4 = comfortable, 8 = just comfortable, 12 = just uncomfortable, 16 = uncomfortable, and 20 = very uncomfortable.

Exercise consisted of treadmill running at 60% VO_2max_ for 60 min within an environmental chamber that was controlled at 35°C and 39% RH. Exercise was monitored to ensure participants maintained the appropriate intensity. Physiological data (body temperatures, expired gases, and HR) were collected at 15 s intervals throughout exercise, then time‐averaged into 5 min intervals to streamline data reporting and improve data interpretation. Due to continuous measurement of expired gases, participants were not allowed to ingest fluids during exercise. After exercise, participants toweled dry and body mass was assessed. The difference in pre‐ and post‐exercise body mass was utilized to calculate sweat rate, which was not corrected for expiratory water loss. A second blood sample was taken immediately following exercise, and participants remained in the laboratory until 1 h after exercise, at which time a third blood sample was taken. The standard equations that were utilized to calculate mean skin temperature, mean body temperature, and physiological strain index (PSI) during exercise (Kenney, 1998; Moran et al., 1998; Ramanathan, 1964) are provided below.

### 2.5 Calculations

Mean skin temperature (*T*sk) was calculated using the following equation (Ramanathan, 1964):

$$T_{\mathrm{sk}}=0.3\;\left(\text{Tchest}+\text{Ttriceps}\right)+0.2\;\left(\text{Tthigh}+0.2\;\text{Tcalf}\right).$$

Mean body temperature (T_b_) was calculated using the following equation (Kenney, 1998):

$$T_{b}=0.8\left(T_{c}\right)+0.2\left(T_{\mathrm{sk}}\right).$$

Physiological strain index (PSI) was calculated using the following equation (Moran et al., 1998):

$$\mathrm{PSI}=5\left(cT_{c}-iT_{c}\right){\left(39.5-iT_{c}\right)}^{-1}+5\left(\mathrm{cHR}-\mathrm{iHR}\right){\left(180-\mathrm{iHR}\right)}^{-1}$$

where c*T*_c_ is current core temperature, i*T*_c_ is initial core temperature, cHR is current heart rate, and iHR is initial heart rate. The PSI ranges from 0 to 10, where 0–2 equals no/little strain; 3–4 equals low strain; 5–6 equals moderate.

### 2.6 Blood collection and analysis

Blood samples were drawn from an antecubital vein using standard venipuncture techniques before exercise (Pre), after exercise (Post), and 1 h after exercise (1‐Post). From these, heparinized blood was centrifuged (Cole Parmer; EW‐17250‐00) at 3000 RCF for 15 min and plasma was aliquoted into sterile 1.7 mL micro‐eppendorf tubes that were frozen at −80°C until batch analysis of NE and 5‐HT, which were analyzed with ELISAs from Thermo Fisher Scientific (Waltham, MA USA) according to manufacturer's instructions. Data were generated on a Synergy HT Microplate Reader from Biotek (Highland Park, Winooski, VT, USA) using Gen5 software. All samples for an individual subject were analyzed on the same plate and protein concentrations were normalized to changes in plasma volume. NE (EEL010, Invitrogen, ThermoFisher Scientific, Waltham, MA, USA) was detectable at 0.31 ng/mL with an intra‐assay coefficient of variation of 8.4% and an inter‐assay coefficient of variation of 3.9%. 5‐HT (EEL006, Invitrogen, ThermoFisher Scientific, Waltham, MA, USA) was detectable at 15.6 ng/mL with an intra‐assay coefficient of variation of 6.1% and an inter‐assay coefficient of variation of 4.6%.

### 2.7 Power analysis

The present human subjects research study was conceived based on prior work in an animal model (Jiang et al., 2013), where berberine was shown to cause large reductions in core temperature [*F* = 25.901, ɳ^2^ = 0.683, Cohen's *D* = 2.94] and heart rate [*F* = 9.168, ɳ^2^ = 0.534, Cohen's *D* = 2.14] in mice. Based on the means and standard deviations reported in that study, with an α level of *p* ≤ 0.05, three subjects would result in an 80% probability of detecting a difference in T_c_ (Jiang et al., 2013) and four subjects would result in an 80% probability of detecting a difference in HR (Jiang et al., 2013). There are differences between animal models and human subject research. For that reason, we also conducted a power analysis (G*Power Version 3.1; Dusseldorf, North Rhine‐Westphalia, Germany) using the means and SDs of prior work that examined short‐term ingestion of a dietary supplement prior to exertional heat stress. From that study (Kuennen et al., 2011) it was determined that with an α level of *p* ≤ 0.05, eight subjects would result in a 90% probability of detecting a difference in T_c_ and a 91% probability of detecting a difference in HR (Kuennen et al., 2011).

### 2.8 Statistical analysis

Statistical analyses were performed using STATISTICA for Windows (version 7.1; StatSoft Inc., Tulsa, OK, USA). Unless stated otherwise, text and table data are presented as mean ± SD for *N* = 8. Two‐tailed paired *t*‐tests were used to determine if dietary intake, urine specific gravity, water ingestion, and sweat rate were different between conditions (Placebo or Berberine). Two‐factor RM‐ANOVAs [where study condition (Placebo or Berberine) and exercise time (0 min through 60 min) served as the repeated measures factors] were used to examine potential differences in VO_2_, VCO_2_, RER, R_R_, V_T_, V_E_, ventilatory equivalents for oxygen consumption (V_E_/VO_2_) and carbon dioxide production (V_E_/VCO_2_), T_c_, T_sk_, T_b_, HR, physiological strain index (PSI), and ratings of thermal sensation, generalized discomfort, and perceived exertion over the 60 min exercise bout. Differences in the absolute change in these variables over the 60 min exercise bout (i.e., delta values) were determined with dependent *t*‐tests. Two‐factor RM‐ANOVAs [where study condition (Placebo or Berberine) and sample timepoint (Pre, Post, and 1‐Post) served as the repeated measures factors] were used to examine potential differences in NE and 5‐HT. Statistical significance was set at *p* ≤ 0.05. Significant main and interaction effects were further evaluated by way of Fisher's LSD post hoc analysis. Effect sizes were calculated as Cohen's d (*d*) for dependent *t*‐tests or as partial eta squared ($\eta_{p}^{2}$) for RM‐ANOVA to provide the reader with an objective indication of the magnitude of difference between groups or variables. For reference, values of 0.2, 0.5, and 0.8 correspond to small, medium, and large effect sizes for *d*, respectively, and values of 0.01, 0.09, and 0.25 are considered to be small, medium, and large effect sizes for $\eta_{p}^{2}$ (Cohen, 1992).

## 3 RESULTS

### 3.1 Equality of study conditions

#### 3.1.1 Ambient conditions

Ambient temperature averaged 35.5 ± 0.9°C in Placebo and 35.6 ± 1.0°C in Berberine. Relative humidity averaged 39.4 ± 2.6% in Placebo and 39.2 ± 4.5% in Berberine. There were no differences in ambient temperature [*F* = 1.180, *p* = 0.313, $\eta_{p}^{2}$ = 0.144] or relative humidity [*F* = 0.177, *p* = 0.687, $\eta_{p}^{2}$ = 0.025] between conditions.

### 3.2 Indirect calorimetry

#### 3.2.1 Oxygen consumption (VO_2_ )

**FIGURE 1:**
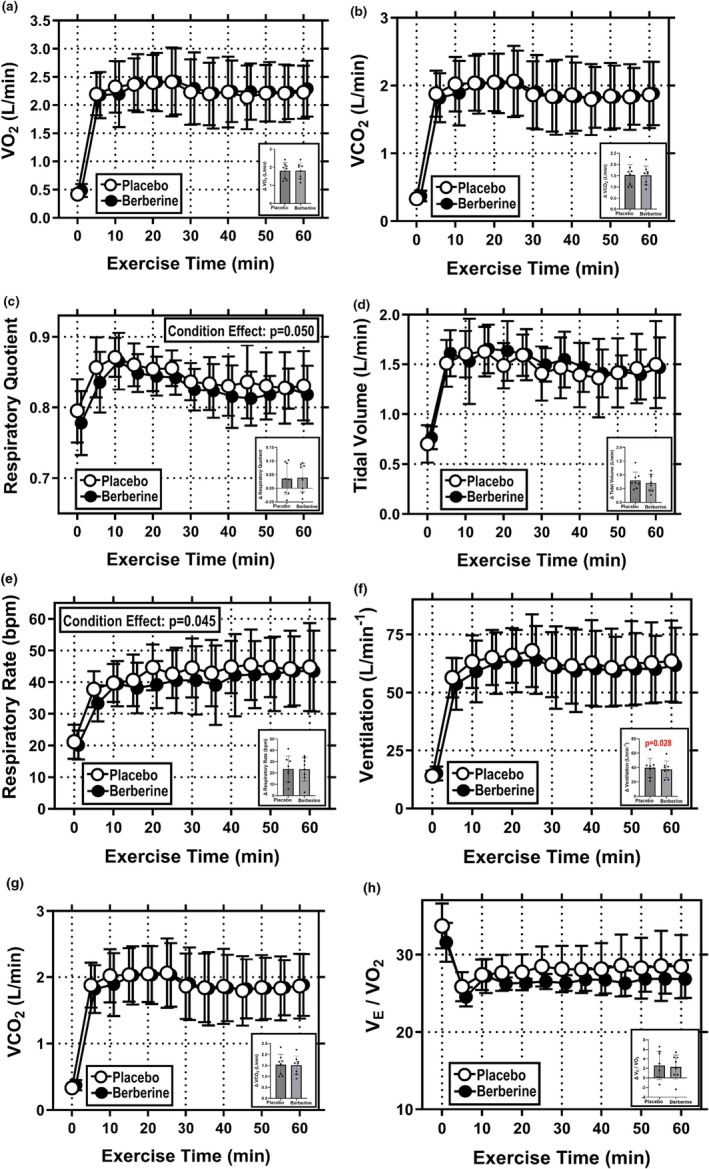
Indirect calorimetry during 60 min of treadmill exercise performed under hot (35°C), moderately humid (39% RH) ambient conditions. (a) oxygen consumption (VO_2_); (b) carbon dioxide production (VCO_2_); (c) respiratory quotient (RQ); (d) respiratory rate (RR); (e) tidal volume (V_T_); (f) minute ventilation; (g) ventilatory equivalent for oxygen consumption (V_E_/VO_2_); and (h) ventilatory equivalent for carbon dioxide production (V_E_/VCO_2_) during 60 min of fixed workload exercise. (a–h) Exercise data at 5 min intervals (main figure) and also as delta values (insets). Data are mean ± SD for *N* = 8. Data were analyzed with two‐way RM ANOVAs, where supplement condition (Placebo or Berberine) and exercise time (0–60 min) served as repeated measure factors. Statistical significance was set at *p* ≤ 0.05. Significant main and interaction effects are identified on individual graphs (where applicable). *Indicates *p* ≤ 0.05 compared with the same timepoint in the opposite study condition.

VO_2_ at the 60 min timepoint averaged 2.23 ± 0.47 L/min in Placebo and 2.29 ± 0.50 L/min in Berberine (Figure 1a). There was no difference in VO_2_ between conditions [*F* = 0.570, *p* = 0.475, $\eta_{p}^{2}$ = 0.075] and the interaction effect of condition and time was not significant [*F* = 1.040, *p* = 0.421, $\eta_{p}^{2}$ = 0.129]. As shown in Figure 1a *inset*, there was also no difference in the absolute rise in VO_2_ over the 60 min exercise bout (ΔVO_2_) in Placebo (1.81 ± 0.44 L/min) and Berberine (1.81 ± 0.43 L/min) [*t* = 0.050, *p* = 0.481, *d* = 0.004].

#### 3.2.2 Carbon dioxide production (VCO_2_ )

VCO_2_ at the 60 min timepoint averaged 1.87 ± 0.49 L/min in Placebo and 1.89 ± 0.47 L/min in Berberine (Figure 1b). There was no difference in VCO_2_ between conditions [*F* = 0.157, *p* = 0.704, $\eta_{p}^{2}$ = 0.022] and the interaction effect of condition and time was not significant [*F* = 0.989, *p* = 0.466, $\eta_{p}^{2}$ = 0.124]. As shown in Figure 1b *inset*, there was also no difference in the absolute rise in VCO_2_ over the 60 min exercise bout (ΔVCO_2_) in Placebo (1.53 ± 0.47 L/min) and Berberine (1.51 ± 0.42 L/min) [*t* = 0.606, *p* = 0.282, *d* = 0.048].

#### 3.2.3 Respiratory exchange ratio (RER)

RER at the 60 min timepoint averaged 0.83 ± 0.05 in Placebo and 0.82 ± 0.04 in Berberine (Figure 1c). There was a significant difference in RER between conditions [*F* = 5.560, *p* = 0.050, $\eta_{p}^{2}$ = 0.443], where RER averaged 0.84 ± 0.04 over the 60 min exercise bout in Placebo and 0.83 ± 0.04 in Berberine. However, as shown in Figure 1c *inset*, there was no difference in the absolute rise in RER over the 60 min exercise bout (ΔRER), which was 0.04 ± 0.06 in Placebo and 0.04 ± 0.05 in Berberine [*t* = −0.273, *p* = 0.396, *d* = −0.068]. Given the small condition effect and the lack of difference in the absolute rise in RER over the 60 min exercise bout, we feel this may be an example of “statistical” but not “practical” significance.

#### 3.2.4 Respiratory rate (R_R_ )

R_R_ at the 60 min timepoint averaged 44.8 ± 13.9 bpm in Placebo and 43.5 ± 12.8 bpm in Berberine (Figure 1d). There was a significant difference in R_R_ between conditions [F = 5.95, *p* = 0.045, $\eta_{p}^{2}$ = 0.460], where R_R_ averaged 41.4 ± 10.6 bpm over the 60 min exercise bout in Placebo and 38.8 ± 11.2 bpm in Berberine. The interaction effect of condition and time was not significant [*F* = 0.841, *p* = 0.609, $\eta_{p}^{2}$ = 0.107]. As shown in Figure 1d *inset*, there was also no difference in the absolute rise in R_R_ over the 60 min exercise bout (ΔR_R_) in Placebo (23.6 ± 11.4 bpm) and Berberine (23.4 ± 10.6 bpm) [*t* = 0.106, *p* = 0.459, *d* = 0.020].

#### 3.2.5 Tidal volume (V_T_ )

V_T_ at the 60 min timepoint averaged 1.50 ± 0.44 L/min in Placebo and 1.47 ± 0.31 L/min in Berberine (Figure 1e). There was no difference in V_T_ between conditions [*F* = 0.426, *p* = 0.535, $\eta_{p}^{2}$ = 0.057] and the interaction effect of condition and time was not significant [*F* = 0.851, *p* = 0.599, $\eta_{p}^{2}$ = 0.108]. As shown in Figure 1e *inset*, there was also no difference in the absolute rise in V_T_ over the 60 min exercise bout (ΔV_T_) in Placebo (0.80 ± 0.31 L/min) and Berberine (0.70 ± 0.31 L/min) [*t* = 1.123, *p* = 0.149, *d* = 0.299].

#### 3.2.6 Minute ventilation (V_E_ )

V_E_ at the 60 min timepoint averaged 63.5 ± 17.4 L/min in Placebo and 61.8 ± 16.2 L/min in Berberine (Figure 1f). There was no difference in V_E_ between conditions [*F* = 2.187, *p* = 0.183, $\eta_{p}^{2}$ = 0.238] and the interaction effect of condition and time was not significant [*F* = 0.713, *p* = 0.735, $\eta_{p}^{2}$ = 0.093]. However, as shown in Figure 1f *inset*, there was a difference in the absolute rise in V_E_ over the 60 min exercise bout (ΔV_E_), which was 49.4 ± 16.7 L/min in Placebo and 46.6 ± 14.3 L/min in Berberine [*t* = 2.36, *p* = 0.028, *d* = 0.179].

#### 3.2.7 Ventilatory equivalent for oxygen consumption (V_E_ /VO_2_ )

V_E_/VO_2_ at the 60 min timepoint averaged 28.5 ± 4.1 L/min in Placebo and 26.8 ± 2.5 L/min in Berberine (Figure 1g). There was a trend towards differences in V_E_/VO_2_ between conditions [*F* = 4.49, *p* = 0.071, $\eta_{p}^{2}$ = 0.391] but the interaction effect of condition and time was not significant [*F* = 0.999, *p* = 0.457, $\eta_{p}^{2}$ = 0.125]. As shown in Figure 1g *inset*, there was also no difference in the absolute rise in V_E_/VO_2_ over the 60 min exercise bout (ΔV_E_/VO_2_) in Placebo (2.60 ± 2.94 L/min) and Berberine (2.29 ± 2.57 L/min) [*t* = 0.695, *p* = 0.255, *d* = 0.111].

#### 3.2.8 Ventilatory equivalent for carbon dioxide production (V_E_ /VCO_2_ )

V_E_/VCO_2_ at the 60 min timepoint averaged 34.3 ± 4.4 L/min in Placebo and 32.8 ± 2.2 L/min in Berberine (Figure 1h). There was no difference in V_E_/VCO_2_ between conditions [*F* = 2.711, *p* = 0.144, $\eta_{p}^{2}$ = 0.279] and the interaction effect of condition and time was not significant [*F* = 1.089, *p* = 0.379, $\eta_{p}^{2}$ = 0.135]. As shown in Figure 1h *inset*, there was also no difference in the absolute rise in V_E_/VCO_2_ over the 60 min exercise bout (Δ V_E_/VCO_2_) in Placebo (4.1 ± 3.3 L/min) and Berberine (3.4 ± 2.4 L/min) [*t* = 1.202, *p* = 0.134, *d* = 0.233].

### 3.3 Thermal and cardiovascular strain

#### 3.3.1 Core temperature (T_c_)

**FIGURE 2:**
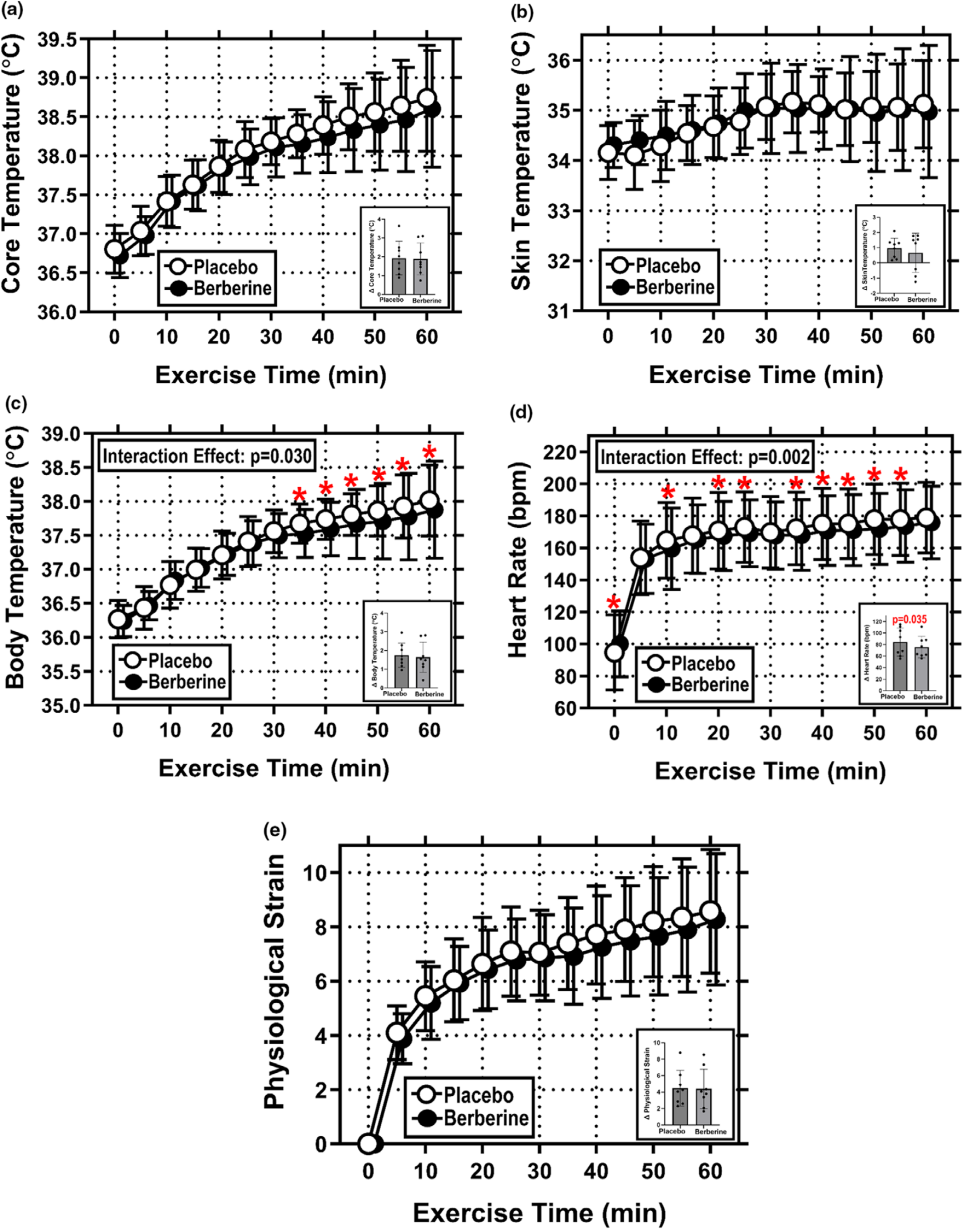
Cardiovascular and thermal strain during 60 min of treadmill exercise performed under hot (35°C), moderately humid (39% RH) ambient conditions. (a) Core temperature; (b) mean skin temperature; (c) mean body temperature; (d) heart rate; and (e) physiological strain index during 60 min of fixed workload exercise. (a–e) Exercise data at 5 min intervals (main figure) and also as delta values (insets). Data are mean ± SD for *N* = 8. Data were analyzed with two‐way RM ANOVAs, where supplement condition (Placebo or Berberine) and exercise time (0–60 min) served as repeated measure factors. Statistical significance was set at *p* ≤ 0.05. Significant main and interaction effects are identified on individual graphs (where applicable). *Indicates *p* ≤ 0.05 compared with the same timepoint in the opposite study condition.

T_c_ at the 60 min timepoint averaged 38.74 ± 0.68°C in Placebo and 38.60 ± 0.75°C in Berberine (Figure 2a). There was no difference in T_c_ between conditions [*F* = 1.20, *p* = 0.306, $\eta_{p}^{2}$ = 0.148] and the interaction effect of condition and time was not significant [*F* = 1.000, *p* = 0.463, $\eta_{p}^{2}$ = 0.124]. As shown in Figure 2a *inset*, there was also no difference in the absolute rise in T_c_ over the 60 min exercise bout (ΔT_c_) in Placebo (1.94 ± 0.90°C) and Berberine (1.88 ± 0.85°C) [*t* = 0.327, *p* = 0.377, *d* = 0.058].

#### 3.3.2 Mean skin temperature (T_sk_)

T_sk_ at the 60 min timepoint averaged 35.12 ± 0.87°C in Placebo and 34.98 ± 1.32°C in Berberine (Figure 2b). There was no difference in T_sk_ between conditions [*F* = 0.050, *p* = 0.832, $\eta_{p}^{2}$ = 0.007] and the interaction effect of condition and time was not significant [*F* = 0.790, *p* = 0.660, $\eta_{p}^{2}$ = 0.101]. As shown in Figure 2b *inset*, there was also no difference in the absolute rise in T_sk_ over the 60 min exercise bout (ΔT_sk_) in Placebo (0.97 ± 0.66°C) and Berberine (0.67 ± 1.29°C) [*t* = 0.733, *p* = 0.244, *d* = 0.288].

#### 3.3.3 Mean body temperature (T_b_)

T_b_ at the 60 min timepoint averaged 38.01 ± 0.52°C in Placebo and 37.88 ± 0.72°C in Berberine (Figure 2c). While there was no difference in T_b_ between conditions [*F* = 1.000, *p* = 0.348, $\eta_{p}^{2}$ = 0.126], the interaction effect of condition and time was significant [*F* = 2.000, *p* = 0.030, $\eta_{p}^{2}$ = 0.226], where T_b_ was lower in Berberine (37.69 ± 0.53°C) than Placebo (37.84 ± 0.38°C) from 35 to 60 min of exercise. However, as shown in Figure 2c *inset*, there was no difference in the absolute rise in T_b_ over the 60 min exercise bout (ΔT_b_) in Placebo (1.75 ± 0.65°C) and Berberine (1.64 ± 0.81°C) [*t* = 0.760, *p* = 0.236, *d* = 0.144].

#### 3.3.4 Heart rate (HR)

HR at the 60 min timepoint averaged 179 ± 22 bpm in Placebo and 176 ± 23 bpm in Berberine (Figure 2d). Both the main effect of condition [*F* = 8.106, *p* = 0.025, $\eta_{p}^{2}$ = 0.537] and the interaction effect of condition and time were significant [*F* = 2.874, *p* = 0.002, $\eta_{p}^{2}$ = 0.291], where HR was lower in Berberine than Placebo at 0, 10, 20, 25, and 35–55 min of exercise. For reference, heart rate over these timepoints averaged 165 ± 33 bpm in Placebo and 161 ± 30 bpm in Berberine. As shown in Figure 2d *inset*, the absolute rise in HR over the 60 min exercise bout (ΔHR) was also different between study conditions, where HR increased 77 ± 22 bpm in Placebo and 69 ± 13 bpm in Berberine [*t* = 2.130, *p* = 0.035, *d* = 0.447].

#### 3.3.5 Physiological strain index (PSI)

PSI at the 60 min timepoint averaged 8.6 ± 2.3 A.U. in Placebo and 8.3 ± 2.4 A.U. in Berberine (Figure 2e). There was no difference in PSI between conditions [*F* = 2.42, *p* = 0.164, $\eta_{p}^{2}$ = 0.257] and the interaction effect of condition and time was not significant [*F* = 0.509, *p* = 0.892, $\eta_{p}^{2}$ = 0.068]. As shown in Figure 2e *inset*, there was also no difference in the absolute rise in PSI over the 60 min exercise bout (ΔPSI) in Placebo (4.5 ± 2.2 A.U.) and Berberine (4.4 ± 2.4 A.U.) [*t* = 0.165, *p* = 0.437, *d* = 0.035].

#### 3.3.6 Hydration

Urine specific gravity (USG) was determined from pre‐exercise urine samples. USG, which averaged 1.019 ± 0.002 in Placebo and 1.021 ± 0.002 in Berberine, was not different between conditions [*t* = −0.606, *p* = 0.282, *d* = −0.281]. Sweat rate, which averaged 1.19 ± 0.15 L/h in Placebo and 1.21 ± 0.13 L/h in Berberine, was not different between conditions [*t* = −0.625, *p* = 0.276, *d* = −0.066].

### 3.4 Perceptual data

#### 3.4.1 Thermal sensation

**FIGURE 3:**
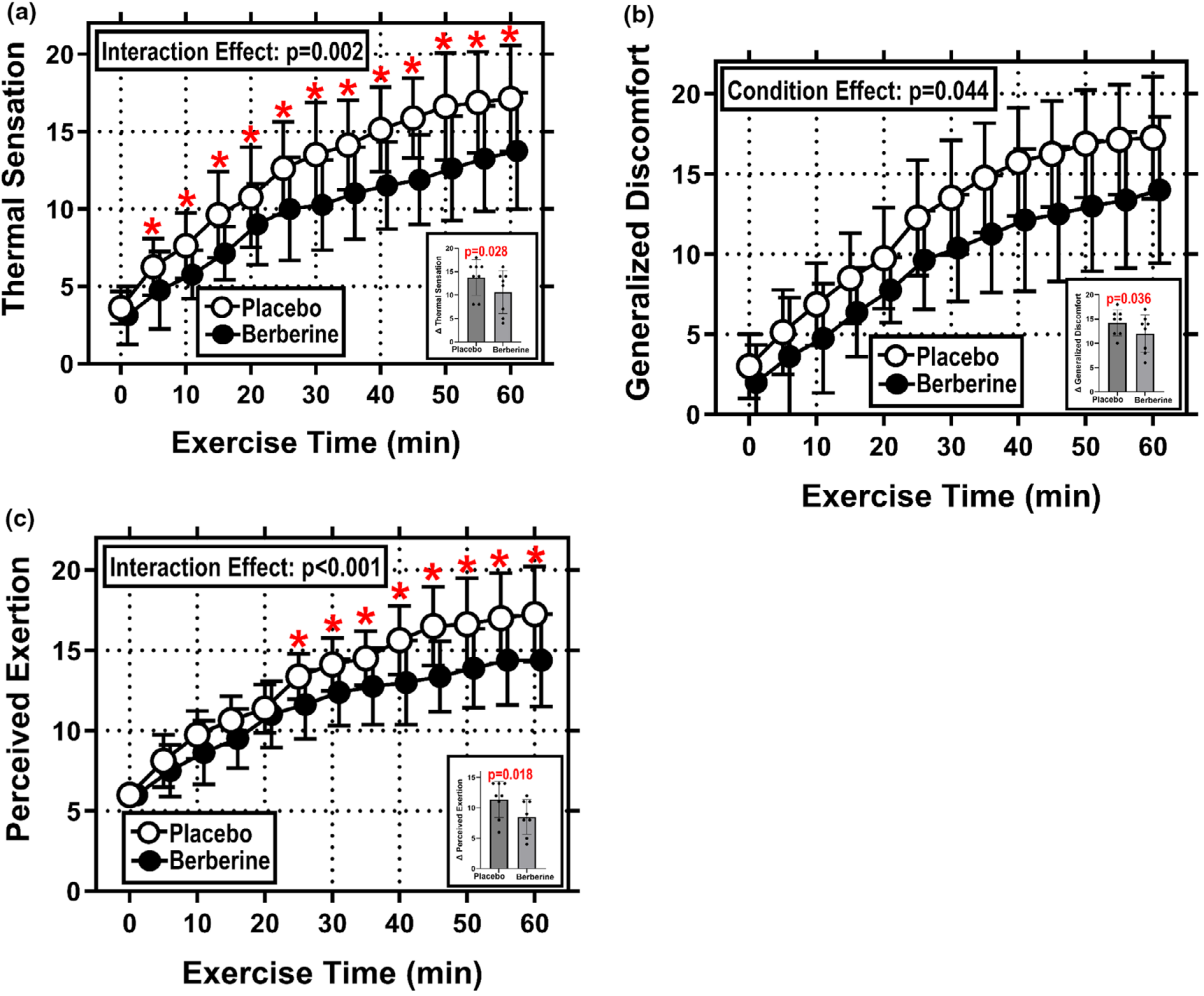
Perceptual responses during 60 min of treadmill exercise performed under hot (35°C), moderately humid (39% RH) ambient conditions. (a) Thermal sensation; (b) generalized discomfort; and (c) rating of perceived exertion during 60 min of fixed workload exercise. (a–c) Exercise data at 5 min intervals (main figure) and also as delta values (insets). Data are mean ± SD for *N* = 8. Data were analyzed with two‐way RM ANOVAs, where supplement condition (Placebo or Berberine) and exercise time (0–60 min) served as repeated measure factors. Statistical significance was set at *p* ≤ 0.05. Significant main and interaction effects are identified on individual graphs (where applicable). *Indicates *p* ≤ 0.05 compared with the same timepoint in the opposite study condition.

Thermal sensation at the 60 min timepoint averaged 17.1 ± 3.4 A.U. in Placebo and 13.8 ± 3.8 A.U. in Berberine (Figure 3a). Both the main effect of condition [*F* = 7.23, *p* = 0.031, $\eta_{p}^{2}$ = 0.507] and the interaction effect of condition and time were significant [*F* = 2.86, *p* = 0.002, $\eta_{p}^{2}$ = 0.291], where thermal sensation was lower in Berberine than Placebo from 5 to 60 min of exercise. For reference, thermal sensation over these timepoints averaged 13.0 ± 4.5 A.U. in Placebo and 10.1 ± 3.9 A.U. in Berberine. As shown in Figure 3a *inset*, there was also a difference in the absolute rise in thermal sensation over the 60 min exercise bout (Δ thermal sensation), which increased 13.8 ± 3.8 A.U. in Placebo and 10.6 ± 4.6 A.U. in Berberine [*t* = 2.284, *p* = 0.028, *d* = 0.743].

#### 3.4.2 Generalized discomfort

Generalized discomfort at the 60 min timepoint averaged 17.3 ± 3.8 A.U. in Placebo and 14.0 ± 4.6 A.U. in Berberine (Figure 3b). The main effect of condition [*F* = 6.009, *p* = 0.044, $\eta_{p}^{2}$ = 0.462] was significant. The interaction effect of condition and time exhibited a trend [*F* = 1.723, *p* = 0.076, $\eta_{p}^{2}$ = 0.198], but was not significant. For reference, generalized discomfort over the 60 min exercise bout averaged 12.1 ± 5.6 A.U. in Placebo and 9.3 ± 5.1 A.U. in Berberine. As shown in Figure 3b *inset*, there was also a difference in the absolute rise in generalized discomfort over the 60 min exercise bout (Δ generalized discomfort), which increased 14.3 ± 2.7 A.U. in Placebo and 12.0 ± 3.9 A.U. in Berberine [*t* = 2.113, *p* = 0.036, *d* = 0.680].

#### 3.4.3 Rating of perceived exertion

Ratings of perceived exertion (RPE) at the 60 min timepoint averaged 17.3 ± 3.0 A.U. in Placebo and 14.4 ± 2.9 A.U. in Berberine (Figure 3c). Both the main effect of condition [*F* = 6.702, *p* = 0.036, $\eta_{p}^{2}$ = 0.489] and the interaction effect of condition and time were significant [*F* = 3.263, *p* < 0.001, $\eta_{p}^{2}$ = 0.318], where RPE was lower in Berberine than Placebo from 25 to 60 min of exercise. For reference, RPE over these timepoints averaged 15.6 ± 2.6 A.U. in Placebo and 13.2 ± 2.5 A.U. in Berberine. As shown in Figure 3c *inset*, there was also a difference in the absolute rise in RPE over the 60 min exercise bout (ΔRPE), which increased 11.4 ± 3.0 A.U. in Placebo and 8.5 ± 2.9 A.U. in Berberine [*t* = 2.592, *p* = 0.018, *d* = 0.983].

#### 3.4.4 Nor‐epinephrine

**FIGURE 4:**
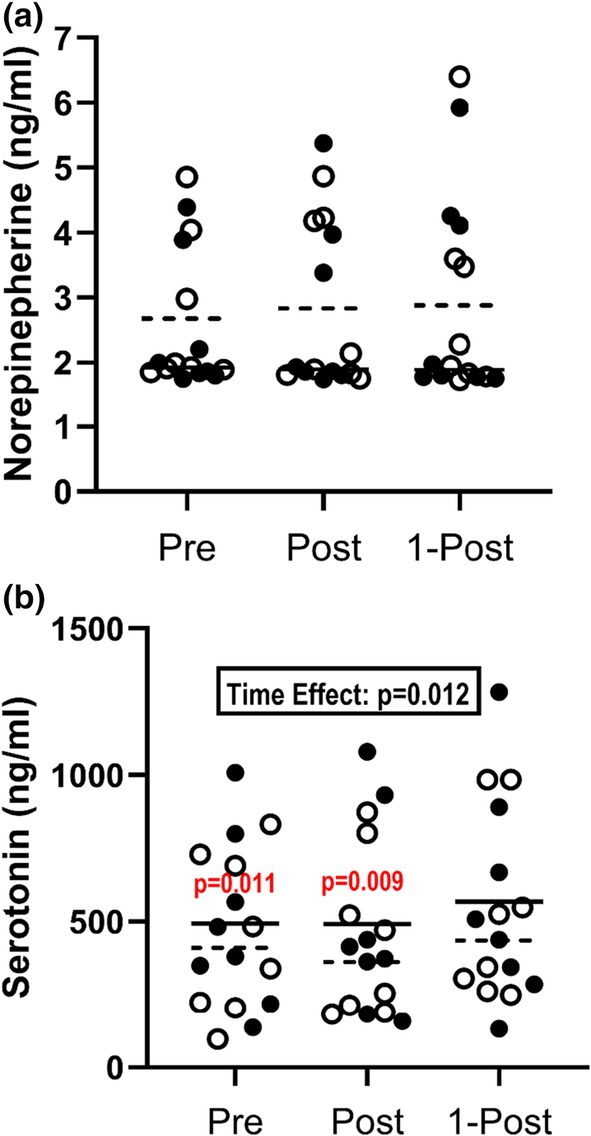
Circulating concentrations of monoamine neurotransmitters during 60 min of treadmill exercise performed under hot (35°C), moderately humid (39% RH) ambient conditions. (a) Nor‐epinephrine and (b) serotonin concentrations in blood samples collected before (Pre), after (Post), and 1‐h after (1‐Post) exercise. Individual dots represent the individual participant concentrations for Placebo (open circles) and Berberine (closed circles). Lines represent the group mean response in Placebo (dotted line) and Berberine (solid line). Data were analyzed with two‐way RM ANOVAs, where supplement condition (Placebo or Berberine) and blood sample timepoint (Pre, Post, and 1‐Post) served as repeated measure factors. Statistical significance was set at *p* ≤ 0.05. The significant main effect of time is identified for serotonin, where Pre and Post values were lower than values at 1‐Post. For serotonin, specific *p* values are provided numerically on the graph.

Neither the main effect of condition [*F* = 0.416, *p* = 0.547, $\eta_{p}^{2}$ = 0.077] nor the main effect of time [*F* = 0.767, *p* = 0.489, $\eta_{p}^{2}$ = 0.133] was significant for NE (Figure 4a). The interaction effect of condition and time was also not significant [*F* = 0.728, *p* = 0.507, $\eta_{p}^{2}$ = 0.127].

#### 3.4.5 Serotonin

The main effect of condition was not significant for 5‐HT [*F* = 0.276, *p* = 0.616, $\eta_{p}^{2}$ = 0.038] but there was a main effect of time [*F* = 6.197, *p* = 0.012, $\eta_{p}^{2}$ = 0.470], wherein 5‐HT concentrations at 1‐POST were shown to be elevated above levels at PRE (+14%, *p* = 0.011) and POST (+15%, *p* = 0.009) (Figure 4b). The interaction effect of condition and time was not significant [*F* = 0.053, *p* = 0.949, $\eta_{p}^{2}$ = 0.007].

## 4 DISCUSSION

The present study investigated the impact of 1 week of 1.5 g daily dietary berberine supplementation on physiological and perceptual responses during 1 h of exercise in hot and moderately humid conditions. Mean body temperature was reduced but core and skin temperatures were not altered. The respiratory quotient was lower following berberine supplementation, but volumes of oxygen consumption and carbon dioxide production were not improved. Heart rate and respiratory rate were reduced and there was a trend towards a reduction in the ventilatory equivalent for oxygen consumption. However, tidal volume and minute ventilation were unchanged. Thus, while there was some evidence of improved physiological function following berberine supplementation, overall our results were mixed. The most significant study findings were participants' perceptual ratings of thermal strain, generalized discomfort, and perceived exertion, which were dramatically improved. Collectively these data indicate that 1 week of 1.5 g/d dietary berberine supplementation may improve select physiologic and perceptual responses during thermally challenging exercise. These findings have practical relevance for endurance athletes, military personnel, wildland fire fighters, and other groups that need to maintain optimal performance during prolonged work or exercise in the heat.

Berberine has been shown to improve core temperature regulation in multiple animal experiments. Jiang reported that 2 h following intravenous injection of saline or berberine at dosages ranging from 0.2 to 0.8 mg/kg berberine injection, saline‐injected mice exhibited a peak core temperature increase of ~2.5°C during passive hyperthermia exposure (40°C for 2 h), whereas berberine injected mice exhibited peak core temperature increases ranging from ~0.5°C to ~1°C (Jiang et al., 2013). Berberine was also shown to reduce the rectal temperature of rats that were maintained under thermoneutral conditions (Kulkarni & Dhir, 2008). A similar dose/response relationship was shown in a rabbit model that examined the impact of intragastric berberine administration (at dosages of 0.04–0.06 g/kg) on the LPS‐mediated fever response. At 1 h post LPS treatment control rabbits exhibited a ~0.95°C elevation in core temperature, whereas rabbits that received intragastric berberine at 40, 50, and 60 mg/kg exhibited ~0.7°C, ~0.67°C, and ~0.65°C elevations in core temperature, respectively (Chu et al., 2014). Another study examined the impact of *T. foliolosum root*, which contains berberine alkaloids, on body temperature elevations in mice that received 15% yeast suspension injections to cause hyperthermia. Oral ingestion of *T. foliolosum root* at a dose of 300–500 mg/kg allowed mice to fully recover from yeast‐mediated hyperthermia by 2 h post injection, which was similar to the fever reduction provided by acetaminophen (Gopalkrishnan, 2014). When taken together, these studies indicate that berberine significantly improves core temperature regulation in animals that are challenged with passive hyperthermia, LPS‐mediated hyperthermia, and yeast‐mediated hyperthermia.

Although there are differences in the thermoeffector responses to fever and exertional heat stress, exercise‐heat stress shares key features with passive heat stress, including elevated tissue temperatures and inflammatory signaling. For example, in the aforementioned passive heat stress model (40°C) (Jiang et al., 2013), not only did berberine antagonize the rise in core body temperature, it also inhibited HSP70 and TNFα expression. As such, a plausible explanation for the improved thermal response in the present study is that transcriptional suppression of HSP70 and TNFα may have attenuated the stimulus for rising core temperature during exercise‐heat stress. In the context of an endotoxin‐mediated fever mechanism, berberine has been shown to antagonize LPS signaling at TLR4/MD‐2, suppressing the induction of NF‐κB and downstream cytokines (IL‐6, TNFα, and IFNβ) (Chu et al., 2014). Therefore, in the event that exertional heat stress increases LPS exposure, berberine's LPS‐antagonist behavior (TLR4/MD‐2) and cytokine suppression could reduce the pyrogenic/inflammatory drive that would otherwise worsen thermal strain. Following oral ingestion, berberine has been shown to rapidly and preferentially accumulate in liver tissues (Tan et al., 2013), which is important because the liver is the largest organ in the reticuloendothelial system and contains the majority (80%–90%) of Kupfer cells (fixed macrophages) that are responsible for clearing LPS from the portal circulation.

However, it is important to point out that in contrast to this prior work in animal models (Chu et al., 2014; Gopalkrishnan, 2014; Jiang et al., 2013; Kulkarni & Dhir, 2008), core temperature responses in exercising humans were not improved in the present study. Differences in our experimental design (active heat stress in healthy humans versus passive heat stress in animals with or without drug exposure) may have contributed to this difference. Importantly, mean body temperature was reduced in the present study, suggesting that berberine may provide a minor benefit during thermally challenging exercise. One possible speculation is that based on the small but statistically significant reduction in RER, berberine may act to increase lipid oxidation during exercise. There is evidence to support this in animal models, where berberine was shown to increase the expression of multiple fatty acid oxidation enzymes and activate the AMPK pathway, resulting in increased levels of PGC‐1α and improved mitochondrial function (Habiba et al., 2025; Rong et al., 2021). Thus, greater reliance on aerobic lipid metabolism could result in fewer metabolites produced in skeletal muscle that stimulate the cardiorespiratory system, which might explain the small change observed. Fat oxidation also yields slightly less energy per unit of oxygen than carbohydrate oxidation. Thus, for the same VO_2_ and external workload, greater reliance on lipid metabolism could result in slightly lower heat production. This mechanism might help to reconcile the pattern of findings that were observed in the study, where we noted a small but statistically significant reduction in mean body temperature in conjunction with improvements in perceptual responses and modest cardiopulmonary changes.

Prior research examining the effect of berberine supplementation on heart rate and oxygen uptake (VO_2_) is sparse and primarily focused on infirmed populations. In congestive heart failure patients, berberine was shown to enhance inotropy and improve hemodynamic responses (cardiac index and left ventricular ejection fraction) (Marin‐Neto et al., 1988). Despite these improvements in myocardial efficiency, which led to reductions in heart rate, VO_2_ was not shown to be reduced (Marin‐Neto et al., 1988). Another congestive heart failure study reported that following 8 weeks of treatment with Placebo or 1.2 g/d Berberine, participants in the Berberine group exhibited an increased (*p* < 0.02) ejection fraction (32 ± 8%) as compared to participants in the Placebo group (27 ± 5%) (Zeng et al., 2003). In vitro models utilizing isolated guinea pig atria have shown that berberine elicits positive inotropic effects via enhancement of the force‐velocity relationship and increased duration of the systolic contraction phase (Shaffer, 1985). Using Yangxinshi tablets, which combine berberine with 12 other active ingredients in a traditional Chinese medicine preparation, another study reported that participants with coronary heart disease who received Yangxinshi tablets exhibited improvements in VO_2_ peak (+0.22 ± 0.28 L/min) as compared to participants that received Placebo (+0.01 ± 0.18 L/min) (Zhang et al., 2022). Whereas in another study on overweight and obese individuals, participants who received 1 g daily berberine in addition to an 8‐week high intensity aerobic exercise intervention were not shown to further improve VO_2_ peak beyond the benefits of exercise alone (Nikseresht et al., 2024).

Marked improvements in perception (thermal sensation, perceived discomfort, and ratings of perceived exertion) were shown following berberine supplementation in the present study. Those improvements were likely related to the reductions in heart rate, respiratory rate, respiratory exchange ratio, and mean body temperature that were outlined above. Alternatively, increased biogenic amine neurotransmitters (NT) could have improved participants' perceptions of effort during exercise heat stress. For example, in a mouse depression model, 2 weeks of berberine (5 mg/kg) administration via intraperitoneal injection was shown to increase 5‐HT and NE levels by 19% and 29%, respectively (Kulkarni & Dhir, 2008). Higher levels of 5‐HT and NE are associated with reduced pain perception, and berberine (10–20 mg/kg) has been shown to reduce pain signals in the cerebral cortex and hippocampus of male Wistar rats that were challenged with reserpine (a potent monoamine NT depletor) (Arora & Chopra, 2013). A human study investigated the impact of bupropion, a dopamine and NE reuptake inhibitor that is used clinically to treat depression, on time trial performance following prolonged cycling (55% Wmax) in warm (30°C) conditions (Watson et al., 2005). Bupropion significantly improved time trial performance, but that benefit came at the cost of elevations in heart rate and core temperature. Despite the increase in these physiological variables, participants that received bupropion reported similar ratings of perceived exertion and thermal sensation during exercise, suggesting that elevated NE concentrations may allow humans to feel better and work harder in hot environments (Watson et al., 2005).

Interestingly, berberine supplementation was not shown to influence NE or 5‐HT concentrations in the present study, indicating changes in these NTs were not responsible for the improvements in participants' perceptions of effort during exercise heat stress. It is also important to point out that in the absence of marked reductions in core temperature and heart rate, greater motivation to perform prolonged work in a hot environment could lead to increased heatstroke risk, so caution may be warranted. Oral berberine supplementation was recently shown to increase dopamine levels and cognitive function in a mouse model of Parkinsons disease via activation of tetrahydrobiopterin producing bacteria in the intestinal microflora (Wang et al., 2021). This is intriguing because the bioavailability of ingested berberine is low (Patel, 2021), suggesting that berberine supplementation may directly stimulate beneficial changes in gastrointestinal physiology in similar fashion to what we have reported following prolonged dietary curcumin supplementation (Szymanski et al., 2018). Further research is warranted in this area.

### 4.1 Limitations

None of the participants reported regular outdoor exercise during the screening process. Participants were asked to maintain their current exercise regimen for study duration. However, data were collected during summer months, so it is possible that if a participant chose to disregard these instructions and began exercising outdoors during the 2‐week washout period this could have contributed to a different heat acclimatization status between the two study conditions. Our 1.5 g daily berberine dose and twice daily supplementation strategy were adopted based on best practices outlined in a meta‐analysis of prior human intervention studies (Dong et al., 2012). Based on the studies that were outlined in that report we estimate that our participants achieved a plasma berberine concentration of 1.5 ng/mL, which is approximately 5 fold above normal baseline measures (Hua et al., 2007; Xu et al., 2020). However, plasma berberine concentrations were not directly measured in the present study, which is a limitation of our findings. The strongest mechanistic evidence for dietary berberine supplementation in the available literature is from passive heat stress and LPS fever models (mice/rabbits), not from direct examinations of exercise‐heat stress. While our first of its kind study in humans does show a similar phenotype (lower mean body temperature/HR) during exertional heat stress, the mechanistic endpoints (e.g., cytokines, HSP70) that are needed to confirm whether this pathway is operational during human exercise‐heat stress were not examined. Future research should be conducted to answer this question.

## 5 CONCLUSION

The present study investigated the impact of 1 week of 1.5 g/d dietary berberine supplementation on physiologic and perceptual changes during 1 h of moderate intensity exercise in hot and moderately humid conditions. Most physiological variables were unchanged and the improvements that were shown were underwhelming. In contrast, perceptual ratings of thermal strain, generalized discomfort, and perceived exertion were all significantly improved. Given the modest benefits that were shown following short term dietary supplementation in the present study, we do not recommend berberine as a stand‐alone dietary supplement for endurance athletes, military soldiers, wildland firefighters, and other groups that are regularly challenged with prolonged work or exercise in the heat. However, in conjunction with other dietary supplements like blackcurrant extract, curcumin, and glutamine, which have more clearly defined benefits (Conrad et al., 2024; Szymanski et al., 2018; Zuhl et al., 2014), berberine may represent a cost‐effective strategy to reduce thermal strain and improve perception during exercise‐heat stress.

## AUTHOR CONTRIBUTIONS

**Dante A. Van Arman:** Data curation; investigation; methodology; visualization. **Jacob C. Saunders:** Data curation; investigation; methodology. **Yaw O. Korankyi:** Data curation; investigation; methodology. **Emerson P. Heckler:** Data curation; investigation; methodology. **Ben J. Lee:** Conceptualization; investigation; methodology; project administration. **Trevor L. Gillum:** Conceptualization; investigation; methodology; project administration. **Matthew R. Kuennen:** Conceptualization; data curation; formal analysis; funding acquisition; investigation; methodology; project administration; resources; software; supervision; validation; visualization.

## FUNDING INFORMATION

Institutional funding was provided by High Point University, Congdon School of Health Sciences.

## CONFLICT OF INTEREST STATEMENT

The authors have no financial or non‐financial interests that are directly or indirectly related to the work submitted for publication.

## ETHICS STATEMENT

The study authors have no conflicts of interest to report.

## Data Availability

Data generated or analyzed during this study are available from the corresponding author upon reasonable request.
