# Correction to “Dietary berberine supplementation improves select physiological and psychological responses during exertional heat stress: A pilot study in young adults”

**DOI:** 10.14814/phy2.71011

**Published:** 2026-07-13

Van Arman, D. A., Saunders, J. C., Korankyi, Y. O., Heckler, E. P., Lee, B. J., Gillum, T. L., & Kuennen, M. R. (2026). Dietary berberine supplementation improves select physiological and psychological responses during exertional heat stress: A pilot study in young adults. *Physiological Reports, 14*(10), e70937. https://doi.org/10.14814/phy2.70937.

In Figure 1, the placement of the panels is incorrect in the original article:

- Figure 1d should be respiratory rate (currently tidal volume).
- Figure 1e should be tidal volume (currently respiratory rate).
- Figure 1g should be V_E_/VO_2_ (currently VCO_2_).
- Figure 1h should be V_E_/VCO_2_ (currently V_E_/VO_2_).

The text in the Results section is correct. However, the hyperlinks that relate to the above errors bring the reader to the wrong panel in Figure 1. The correct figure is as follows:

**Figure:**
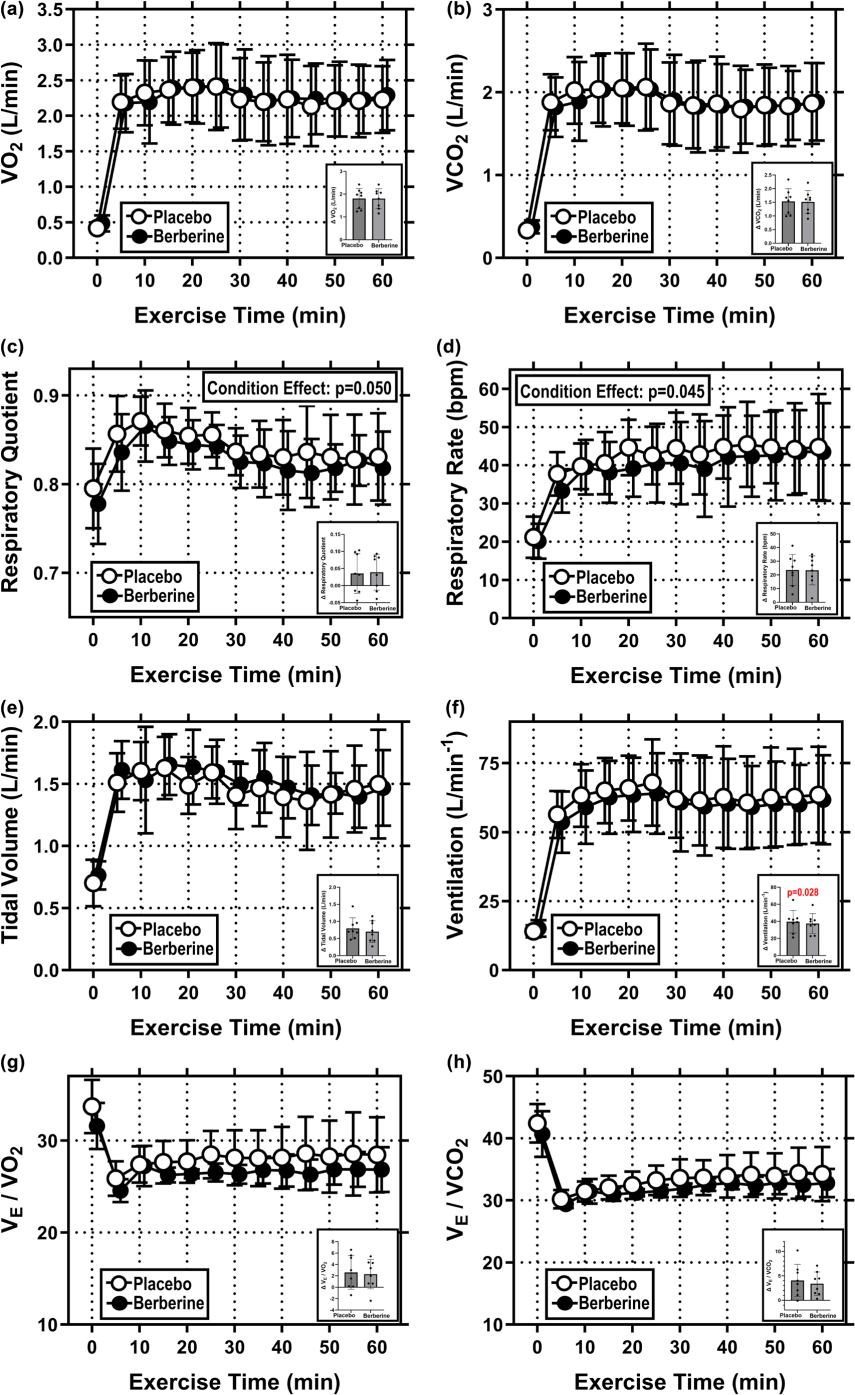
Fig 1. Indirect calorimetry during 60 min of treadmill exercise performed under hot (35°C), moderately humid (39% RH) ambient conditions. (A) oxygen consumption (VO_2_); (B) carbon dioxide production (VCO_2_); (C) respiratory quotient (RQ); (D) respiratory rate (RR); (E) tidal volume (VT); (F) minute ventilation; (G) ventilatory equivalent for oxygen consumption (VE/VO_2_); and (H) ventilatory equivalent for carbon dioxide production (VE/VCO_2_) during 60 min of fixed workload exercise. A–H: Exercise data at 5‐min intervals (main figure) and also as delta values (insets). Data are mean ± SD for *N* = 8. Data were analyzed with two‐way RM ANOVAs, where supplement condition (placebo or berberine) and exercise time (0–60 min) served as repeated measure factors. Statistical significance was set at *p* < 0.05. Significant main and interaction effects are identified on individual graphs (where applicable). * indicates *p* < 0.05 compared with the same timepoint in the opposite study condition.

The authors apologize for these errors, which do not affect the scientific conclusions of the study.

